# Impaired prefrontal synaptic gain in people with psychosis and their relatives during the mismatch negativity

**DOI:** 10.1002/hbm.23035

**Published:** 2015-10-27

**Authors:** Siri Ranlund, Rick A. Adams, Álvaro Díez, Miguel Constante, Anirban Dutt, Mei‐Hua Hall, Amparo Maestro Carbayo, Colm McDonald, Sabrina Petrella, Katja Schulze, Madiha Shaikh, Muriel Walshe, Karl Friston, Dimitris Pinotsis, Elvira Bramon

**Affiliations:** ^1^ Division of Psychiatry University College London London United Kingdom; ^2^ Institute of Cognitive Neuroscience, University College London London United Kingdom; ^3^ Department of Psychiatry Hospital Beatriz Angelo Lisbon Portugal; ^4^ The South London and Maudsley NHS Foundation Trust NIHR Biomedical Research Centre for Mental Health at the Institute of Psychiatry, Psychology and Neuroscience, King's College London United Kingdom; ^5^ Psychology Research Laboratory Harvard Medical School, McLean Hospital Belmont Massachusetts USA; ^6^ Department of Psychiatry Clinical Science Institute, National University of Ireland Galway Ireland; ^7^ Department of Psychiatry Clinical and Experimental Science Institute, University of Foggia Italy; ^8^ The South London and Maudsley NHS Foundation Trust University Hospital Lewisham London United Kingdom; ^9^ Neuroepidemiology and Ageing Research Unit, Imperial College London United Kingdom; ^10^ The Wellcome Trust Centre for Neuroimaging, Institute of Neurology, University College London London United Kingdom

**Keywords:** psychosis, schizophrenia, unaffected relatives, genetic risk, effective connectivity, dynamic causal modeling, DCM, cortical excitability, cortical gain, NMDA receptor

## Abstract

The mismatch negativity (MMN) evoked potential, a preattentive brain response to a discriminable change in auditory stimulation, is significantly reduced in psychosis. Glutamatergic theories of psychosis propose that hypofunction of NMDA receptors (on pyramidal cells and inhibitory interneurons) causes a loss of synaptic gain control. We measured changes in neuronal effective connectivity underlying the MMN using dynamic causal modeling (DCM), where the gain (excitability) of superficial pyramidal cells is explicitly parameterised. EEG data were obtained during a MMN task—for 24 patients with psychosis, 25 of their first‐degree unaffected relatives, and 35 controls—and DCM was used to estimate the excitability (modeled as self‐inhibition) of (source‐specific) superficial pyramidal populations. The MMN sources, based on previous research, included primary and secondary auditory cortices, and the right inferior frontal gyrus. Both patients with psychosis and unaffected relatives (to a lesser degree) showed increased excitability in right inferior frontal gyrus across task conditions, compared to controls. Furthermore, in the same region, both patients and their relatives showed a reversal of the normal response to deviant stimuli; that is, a decrease in excitability in comparison to standard conditions. Our results suggest that psychosis and genetic risk for the illness are associated with both context‐dependent (condition‐specific) and context‐independent abnormalities of the excitability of superficial pyramidal cell populations in the MMN paradigm. These abnormalities could relate to NMDA receptor hypofunction on both pyramidal cells and inhibitory interneurons, and appear to be linked to the genetic aetiology of the illness, thereby constituting potential endophenotypes for psychosis. *Hum Brain Mapp 37:351–365, 2016*. © **2015 The Authors Human Brain Mapping Published by Wiley Periodicals, Inc.**

## INTRODUCTION

Psychotic disorders are among the most severe and enduring mental illnesses, characterised by a distorted sense of reality; an inability to distinguish subjective experiences from the objective world. Disorders where psychosis is commonly experienced include, amongst others, schizophrenia, bipolar disorder, and schizoaffective disorder [NICE, 2014; WHO, 2008].

The mismatch negativity (MMN) event related potential is a pre‐attentive brain response to a discriminable change in auditory stimulation [Duncan et al., 2009; Näätänen, 1992; Todd et al., 2013; Umbricht et al., 2005]. Reduced MMN amplitude is one of the most reliable findings in schizophrenia research, and since the first publication by Shelley et al [1991] over 100 papers have commented on this reduced amplitude [e.g., Baldeweg and Hirsch, 2015; Shaikh et al., 2012; Todd et al., 2013], with a mean effect size of 0.99 [Umbricht et al., 2005]. The MMN is abnormal in clinical risk groups as well as in patients, and is a promising biomarker for psychosis prediction [Bodatsch et al., 2014; Nagai et al., 2013]. Furthermore, the MMN has been proposed as a potential endophenotype or a biological marker of genetic risk for psychosis, because it is heritable [Hall et al., 2006, 2009; Hong et al., 2012], and abnormal in first degree relatives of patients, who have an increased genetic risk for psychosis [Jessen et al., 2001; Michie et al., 2002]. However, not all studies in unaffected relatives have found MMN abnormalities [Bramon et al., 2004; Hong et al., 2012; Kim et al., 2014].

Most previous studies of the MMN use classical electroencephalogram (EEG) analysis methods that investigate the observed amplitude of the event related potential at the sensor level. However, abnormal functional integration among brain regions or “dysconnection,” has been proposed as a core pathology of psychosis [Friston, 1998; Stephan et al., 2006]. Motivated by this hypothesis, we investigated the MMN in terms of the underlying neuronal connectivity. We used dynamic causal modeling (DCM), which explains EEG data using a hierarchical network of dynamically coupled sources, and estimates effective connectivity—the influence that one neuronal system exerts over another—using Bayesian model comparison and inversion [David et al., 2006; Friston et al., 2003]. Several previous DCM studies have found abnormal effective connectivity in psychosis, both using EEG/MEG [Dima et al., 2012, 2010; Fogelson et al., 2014; Roiser et al., 2013] and fMRI methods [Crossley et al., 2009; Deserno et al., 2012; Dima et al., 2009; Mechelli et al., 2007; Schmidt et al., 2014]. However, this is the first DCM study investigating the MMN paradigm in patients as well their unaffected relatives, with a view to examining whether abnormal effective connectivity (and its modulation) could act as an endophenotype for psychosis.

Our hypothesis is based on current theories of psychosis that implicate the neuromodulation of postsynaptic excitability or cortical gain control [Harrison et al., 2011; Lisman et al., 2008; Phillips and Silverstein, 2013; Stephan et al., 2006]. The most ubiquitous neurotransmitter receptor involved in gain modulation is the glutamatergic N‐methyl‐D‐aspartate receptor (NMDA‐R), which is expressed more densely in superficial cortical layers [Friston, 1998; Gonzalez‐Burgos and Lewis, 2012; Lakhan et al., 2013]. NMDA‐R hypofunction is known to be associated with psychosis; it is for example well established that NMDA‐R antagonists such as ketamine or phencyclidine produce psychotomimetic symptoms in healthy individuals and worsen symptoms in patients with schizophrenia [Gilmour et al., 2012; Javitt and Zukin, 1991; Kantrowitz and Javitt, 2010; Krystal et al., 1994; Lahti et al., 1995; Malhotra et al., 1996; Pilowsky et al., 2006]. Recent genetic association studies also implicate the NMDA‐R and its postsynaptic signaling cascade in the disorder [Purcell et al., 2014; Ripke et al., 2014]. Furthermore, the hypofunctioning of NMDA‐Rs on inhibitory GABAergic interneurons is also thought to contribute to a loss of balance between excitation and inhibition, which has been implicated in the neuropathology of psychosis [Gonzalez‐Burgos and Lewis, 2012]. Lastly, reduced MMN amplitudes have been observed in healthy volunteers after NMDA‐R blockade, for example by administration of ketamine [Javitt et al., 1996; Näätänen et al., 2012; Schmidt et al., 2012a; Umbricht et al., 2000]. From a theoretical perspective, this loss of gain control or excitation‐inhibition balance fits comfortably with hierarchical predictive coding models of psychosis and false inference–that rest on the abnormal encoding of uncertainty or precision by the gain of (superficial pyramidal) cells reporting prediction errors [Adams et al., 2013].

Given the prominence of NMDA‐Rs in superficial cortical layers, it is unsurprising that the gain of superficial pyramidal cell populations is strongly affected by NMDA‐R function [Fox et al., 1990; Pinotsis et al., 2014]. In DCM, this gain is parameterized as the inhibitory self‐connectivity (or “intrinsic connectivity”) of superficial pyramidal cells within a cortical source [Friston, 2008]. Our aim in this study was to investigate group differences in MMN responses of patients with psychosis, their unaffected relatives, and healthy controls, and test whether these are best explained by modulations of synaptic gain at different levels of the cortical hierarchy. We hypothesised that, compared to controls, we would see abnormal cortical gain control in both individuals with psychosis and (to a lesser extent) in their first degree relatives.

## MATERIALS AND METHODS

### Sample and Clinical Assessment

The total sample of 84 participants included 24 patients with a psychotic illness (75% schizophrenia, no comorbid diagnoses; see breakdown in Table 1), 25 unaffected first degree relatives of psychosis sufferers (without any personal history of a psychotic illness), and 35 unrelated controls (without any personal or family history of psychotic illnesses).

**Table 1:** Sample demographics (*N* = 84)

	Patients with psychosis *N* = 24	Unaffected relatives *N* = 25	Controls *N* = 35
Mean age (years, SD)	34.6 (±9.3)	43.7 (±14.5)	41.8 (±14.5)
Age range (years)	23–54	16–62	19–69
Gender (*N* male/female, % female)	18/6 (25%)	12/13 (52%)	17/18 (51%)
Education (mean years, SD)	13.6 (±2.8)	14.0 (±3.1)	14.4 (±3.7)
Diagnosis (*N*, %)			
Schizophrenia	18 (75%)	–	–
Schizoaffective disorder	3 (13%)	–	–
Psychosis NOS	1 (4%)	–	–
Bipolar I disorder (w. psychosis)	2 (8%)	–	–
Major Depression	–	3 (12%)	1 (3%)
No psychiatric illness	–	22 (88%)	34 (97%)
Illness duration (mean years, SD)	12.1 (8.4)	NA	NA
Psychotropic medication (*N*, %)	23 (95.8%)	NA	NA
CPZ equivalent (mean, min‐max)*	549.4 (30‐1100)	NA	NA
Years medicated (mean, SD)	10.6 (±8.6)	NA	NA
First medicated (mean years, SD)	24.4 (±7.2)	NA	NA
PANSS (mean, SD)**			
Positive	12.5 (±4.6)	7.2 (±0.6)	7.0 (±0.0)
Negative	14.9 (±5.5)	7.2 (±0.6)	7.0 (±0.0)
General	24.3 (±4.9)	17.5 (±2.0)	16.1 (±0.4)
Relationship to proband (*N*, %)			
Mother	NA	4 (16.0%)	NA
Father	NA	9 (36.0%)	NA
Sister	NA	8 (32.0%)	NA
Brother	NA	3 (12.0%)	NA
Daughter	NA	1 (4.0%)	NA

NA = not applicable; SD = standard deviation; NOS = not otherwise specified; * CPZ equivalent = average chlorpromazine equivalent dosage (mg) for those taking antipsychotic medication (*N* = 18); ** PANSS positive and negative scores range from 7 to 49, PANSS general scores range from 16 to 112

A personal history of nonpsychotic psychiatric illnesses did not constitute an exclusion criterion for relatives or controls, provided they were well and not taking any psychotropic medication at the time of testing and for the preceding 12 months. This was to avoid recruiting biased control groups, unrepresentative of the general and local populations. Three relatives (12%) and one control (3%) had a history of major depressive disorder.

Patients with psychosis and relatives were recruited through voluntary organisations, advertisements in the local press and from clinical teams at the South London and Maudsley NHS Foundation Trust. Controls were recruited by advertisements in the local press and job centres. Participants were excluded if they had a diagnosis of alcohol or substance dependence in the last 12 months, neurological disorders or a previous head injury with loss of consciousness longer than a few minutes.

All participants were clinically interviewed to confirm or exclude a Diagnostic and Statistical Manual of Mental Disorders, Fourth Edition [DSM‐IV; APA, 1994] diagnosis. Instruments used included the Schedule for Affective Disorders and Schizophrenia—Lifetime version [SADS‐L; Endicott and Spitzer, 1978] and the Positive and Negative Syndrome Scale [PANSS; Kay et al., 1987]. Information regarding psychiatric diagnoses of family members not directly assessed was collected from the most reliable informant(s) with the Family Interview for Genetic Studies [FIGS; Maxwell, 1992].

All participants gave informed written consent to participate, and the study was approved by the Institute of Psychiatry Research Ethics Committee, conforming to the standards set by the Declaration of Helsinki. This sample is part of the larger Maudsley Family Study of Psychosis [e.g., Dutt et al., 2012; Ranlund et al., 2014; Schulze et al., 2008; Shaikh et al., 2013].

### EEG Data Acquisition

Electroencephalogram (EEG) was collected from 17 scalp sites according to the 10/20 International system (FP1, FP2, F7, F8, F3, F4, C3, C4, P3, P4, FZ, CZ, PZ, T3, T4, T5, T6), grounded at Fpz using silver/silver‐chloride electrodes [Jasper, 1958]. Vertical, horizontal, and radial electro‐oculographs monitored eye movements, and the left ear lobe served as reference. Data were continuously digitised at 500 Hz with a 0.03–120 Hz band‐pass filter (24 dB/octave roll‐off). Impedances were kept below 5 kΩ [Bramon et al., 2004, 2005].

#### MMN paradigm

This was a duration‐deviant auditory two tone paradigm. The stimuli were 1,200 tones (80 dB, 1,000 Hz, 5 ms rise/fall time), with a 300 ms inter‐stimulus interval, presented in three blocks of 400 stimuli through bilateral intra‐aural earphones. 85% of the tones were “standards” (25 ms duration), and 15% were “deviants” (50 ms duration) [Hall et al., 2009; Shaikh et al., 2012]. The total duration of the experiment was about 10 min.

Participants were sitting comfortably in an armchair, and were instructed to keep their eyes open, fixate on a point in front of them, and disregard the sounds presented.

The classical group comparisons of the MMN amplitude in this sample have been reported in a previous study [Bramon et al., 2004]. Here we undertake a new analysis of effective connectivity during the MMN task.

### EEG Data Preprocessing

Signal processing was conducted using SPM 12b (http://www.fil.ion.ucl.ac.uk/spm/software/spm12) [Litvak et al., 2011] and FieldTrip (http://www.fieldtrip.nl) [Oostenveld et al., 2011] in MATLAB R2013b (http://www.mathworks.co.uk).

The raw EEG data were converted to SPM format, and re‐referenced to the common average. A high‐pass filter of 0.5 Hz was applied, followed by a low‐pass 70 Hz filter. A stop‐pass (49–50 Hz) filter was also applied, to remove line noise. The data were then downsampled to 200 Hz, and epoched with a peristimulus window of −100 to 300 ms. Baseline correction was performed using the 100 ms before stimulus onset.

Independent Component Analysis was used to correct for ocular artefacts in the data. The EEG activity was decomposed into 17 independent components, of which a maximum of two that clearly corresponded to eye blinks were removed from the data. Additional automatic artefact rejection was then conducted, removing any trials whose activity exceeded ±70 μV across all channels. This resulted in an average of 45 trials (3.7%) being rejected per participant, which did not differ between the three groups (*F*(2,81)=1.1, *P =* 0.3).

The EEG data were then averaged using robust averaging in SPM. This procedure produces the best estimate of the average by weighting data points as a function of their distance from the sample mean, so that outlier values have less influence on the overall mean [Wager et al., 2005]. This was followed by an additional low‐pass filter of 70 Hz, as recommended with robust averaging [Litvak et al., 2011].

The grand average event related potential waveforms across subjects were computed for patients, relatives and controls separately. The use of grand average waveforms ensures cleaner (almost noiseless) data for each group and condition. Grand averages retain features that are conserved within groups, and suppress individual differences. These grand averages constitute six event related potentials—one for each group and stimulus condition (standard and deviant tones)—that were characterised in the subsequent DCM analysis [Fogelson et al., 2014].

### Dynamic Causal Modeling

Dynamic causal modeling (DCM) explains measured data using a hierarchical network of dynamically interacting sources, and estimates effective connectivity (the influence that one neuronal system exerts over another), using Bayesian model inversion [Friston et al., 2007]. DCM was originally developed for fMRI [Friston et al., 2003] and was subsequently generalised to other modalities, including evoked responses measured by EEG [David et al., 2006].

DCM permits source reconstruction whilst incorporating biological constraints on neuronal dynamics and coupling [David et al., 2005; Kiebel et al., 2009; Pinotsis et al., 2012]. The neuronal model makes predictions about the dynamics of each source based on the underlying anatomy and biology. We used the canonical microcircuit neural mass model [Bastos et al., 2012], in which each neural source comprises four cell populations: Superficial and deep pyramidal cells, spiny stellate cells and inhibitory interneurons. Each source is connected to other sources via extrinsic excitatory connections, and cell populations within sources are connected to each other via intrinsic connections [Pinotsis et al., 2013]. In this study, we focused on the self‐inhibition of superficial pyramidal cell populations (see Supporting Information Fig. S1), because the strength of this connection reflects the gain (or excitability) of this population, which is linked to NMDA‐R function.

Each source (i.e., each node in the network) was modeled with a single equivalent current dipole under bilateral symmetry assumptions [Kiebel et al., 2006]. We used a boundary elements head model [Fuchs et al., 2001] to approximate the brain, cerebrospinal fluid, skull and scalp surfaces. A canonical MRI head model was used, and coregistration of electrode positions and head model was performed for each subject to map the Montreal Neurological Institute coordinates to points on the head.

Following standard practice, the EEG data were projected onto eight spatial modes to ensure more robust model inversion and dynamical stability. These are the eight principal components or modes of the prior predictive covariance in sensor space [Fastenrath et al., 2009]. We modeled responses from 0 to 250 ms post stimulus onset, to ensure selective modeling of the MMN response *per se*, rather than later components [Garrido et al., 2008].

#### DCM specification

In DCM, Bayesian inference is used to optimise neural source dipoles based on a priori information about their locations. This information is available from studies investigating the sources underlying the MMN—using fMRI [Molholm et al., 2005; Rinne et al., 2005; Schönwiesner et al., 2007], PET [Dittmann‐Balçar et al., 2001; Müller et al., 2002], EEG/MEG [Deouell et al., 1998; Fulham et al., 2014; Jemel et al., 2002; Rinne et al., 2000; Tiitinen et al., 2006], and DCM [Garrido et al., 2007, 2009a, 2008]—showing that the MMN is generated by temporal and frontal sources. Using DCM, the model with the most evidence consists of a three‐level hierarchy comprising bilateral primary auditory cortices (Heschl's gyrus, A1), bilateral superior temporal gyri (STG), and the right inferior frontal gyrus (rIFG). The frontal source is lateralised to the right hemisphere for auditory paradigms [Garrido et al., 2009a; Levanen et al., 1996].

**Figure 1:**
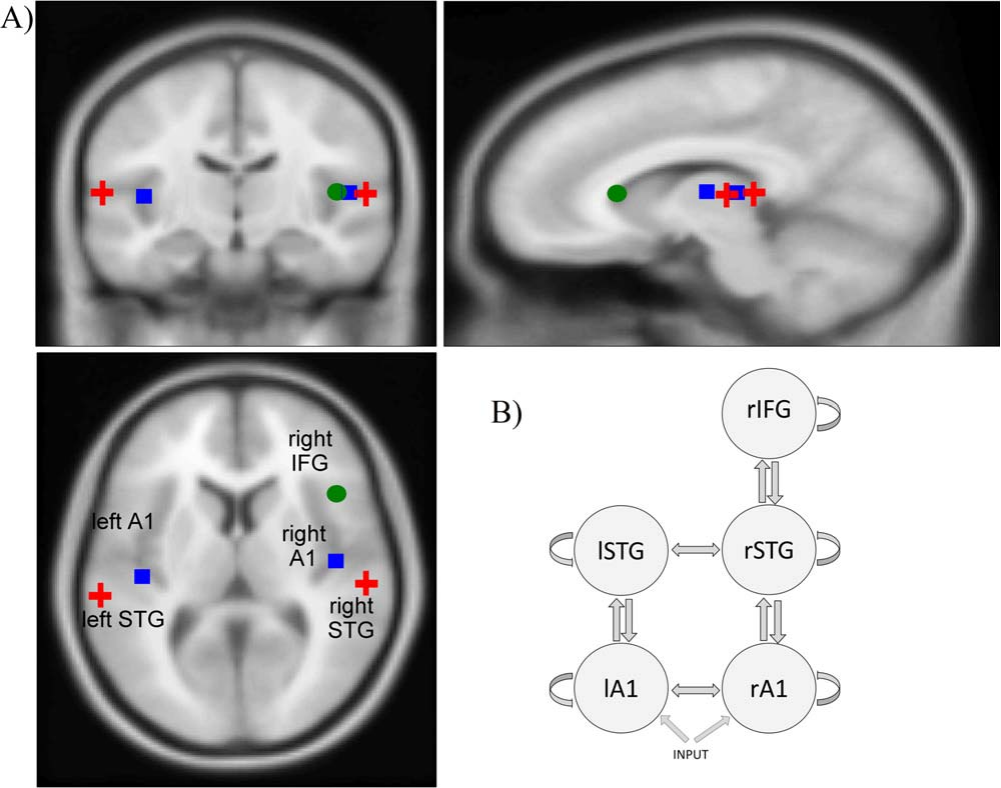
Image showing (A) the prior source locations (overlaid on an MRI image of a standard brain) and (B) the structural model used for dynamic causal modeling. The sources are linked by extrinsic (forward, backward, and lateral) connections, and each source has intrinsic inhibitory self‐connections. A1 = primary auditory cortex; STG = superior temporal gyrus; IFG = inferior frontal gyrus; l = left hemisphere; r = right hemisphere. [Color figure can be viewed in the online issue, which is available at http://wileyonlinelibrary.com.]

Following Garrido et al. [2008], we included the following five sources, with prior source locations in our DCM analysis (in Montreal Neurological Institute coordinates): Left A1 (−42, −22, 7), right A1 (46, −14, 8), left STG (−61, −32, 8), right STG (59, −25, 8), and right IFG (46, 20, 8), illustrated in Figure 1A. DCM incorporates source reconstruction, and the inversion algorithm provides efficient Bayesian estimates of dipole sources that optimise these [David et al., 2005; Kiebel et al., 2009].

Our DCM assumes the existence of extrinsic (forward and backward) connections between, and intrinsic (interlaminar and intralaminar) connections within the specified sources. This has been supported by previous MMN research [Dietz et al., 2014; Garrido et al., 2007, 2009a, 2008]. We also included lateral connections linking left and right A1 and STG [Schmidt et al., 2012b]. Auditory stimuli were modeled as direct input, entering bilateral A1. This model is shown in Figure 1B.

#### Experimental effects

We used condition‐specific grand averaged data over all subjects within each group, allowing us to test for the effect of group directly, as well as the effect of condition by group interactions [e.g., Fogelson et al., 2014; Kiebel et al., 2007]. In other words, the grand averages were treated as the six cells of a 2 × 3 factorial design, with two levels of “condition” (standard and deviant tones) and three levels of “group” (controls, relatives and patients with psychosis).

Group effects were defined as (i) having a genetic risk for psychosis (controls versus relatives and patients combined) and (ii) having a diagnosis of a psychotic illness, irrespective of genetic risk (relatives versus patients). We tested for a main effect of diagnosis and genetic risk on effective connectivity, and the interactions with the effect of condition (standard versus deviant tones). The interactions reflect a diagnosis or risk effect on deviant‐related changes in effective connectivity or postsynaptic sensitivity.

Bayesian model selection was used to find the model with the largest (free energy approximation to the) log model evidence, among the models tested, where models are penalised for increased complexity [Penny et al., 2004]. A difference in log evidence of three or more is considered strong evidence in favour of a model, corresponding to an odds ratio of about 20:1 [Friston and Penny, 2011].

Before testing for the effects of genetic risk and diagnosis, we established the best model to explain the effect of the deviant stimulus across all three groups. We considered eight candidate models with modulations of forward, backward and/or intrinsic connections. The model that allowed for modulations of intrinsic connections (self‐inhibition of superficial pyramidal populations) only had the highest evidence, and was used in all subsequent analyses (see Supporting Information Figs. S2 and S3).

**Figure 2:**
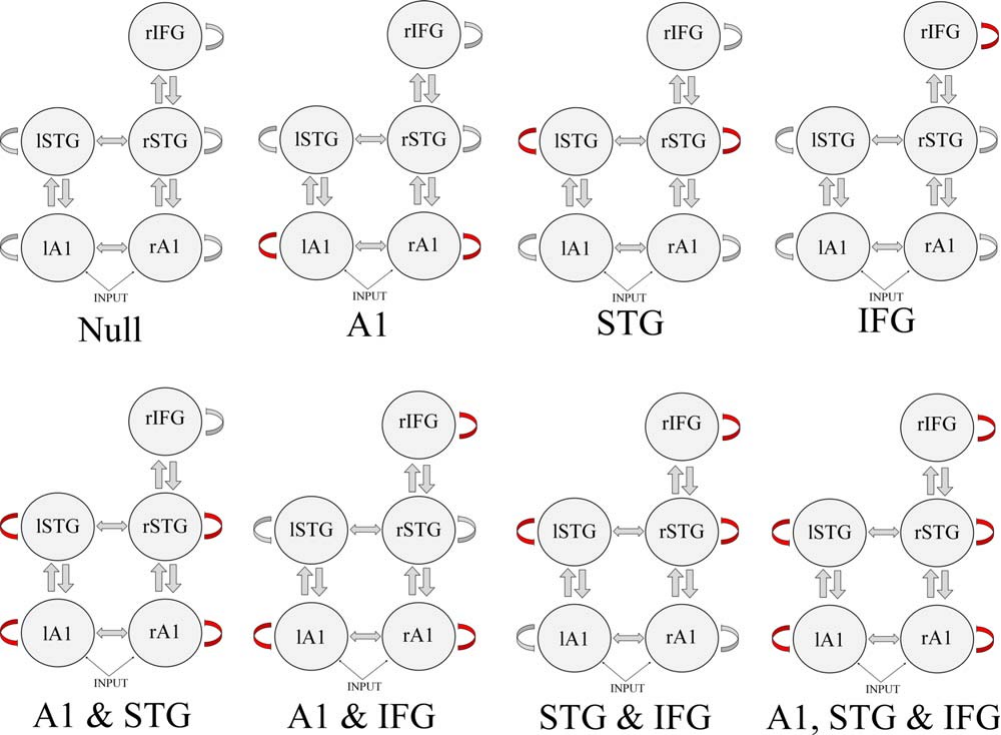
Dynamic causal modeling model space; identifying group differences in intrinsic (self‐inhibitory) connectivity. Red arrows indicate a modulated connection. A1 = primary auditory cortex; STG = superior temporal gyrus; IFG = inferior frontal gyrus; l = left hemisphere; r = right hemisphere. [Color figure can be viewed in the online issue, which is available at http://wileyonlinelibrary.com.]

To study the effects of genetic risk and diagnosis we used Bayesian model selection to establish where in the hierarchy synaptic gain—intrinsic (self‐inhibitory) connectivity—was modulated. Our model space consisted of models with modulations of intrinsic connections at each of the hierarchical levels (A1, STG, rIFG), and all combinations of these. A total of 8 models were thus compared, shown in Figure 2.

Having established the model with the greatest evidence, we examined the posterior estimates of the effective connectivity under this model [Friston and Penny, 2011]. We focused on changes in intrinsic connectivity induced by the mismatch negativity, to identify any differences between patients with psychosis, unaffected relatives, and controls.

## RESULTS

### Sample Demographics

The demographic and clinical characteristics of the sample are detailed in Table 1. All participants were of European Caucasian ethnicity. Patients were significantly younger than controls (*t* = 2.14, *P* = 0.04) and relatives (*t* = 2.60, *P* = 0.01), and this group also contained more males compared to controls (*χ* ^2^ = 4.1, *P* = 0.04) and relatives (*χ* ^2^ = 3.8, *P* = 0.05). Controls and relatives did not differ significantly in age (*t* = 0.51, *P* = 0.61) or gender (*χ* ^2^ = 0.002, *P* = 0.97) distributions. Importantly, patients and relatives together (i.e., the genetic risk group) did not differ from controls in age (*t* = −0.83, *P* = 0.41) or gender (*χ* ^2^ = 1.33, *P* = 0.27) distributions. Years in education did not differ between groups (*F* = 0.40, *P* = 0.67).

The sample comprised 63 families, each including between 1 and 4 individuals. 49 participants (58.3%) were singletons, 18 (21.4%) were part of families with two members in the study, 9 (10.7%) were in three‐person families, and 8 (9.5%) were part of families with four members participating. All unaffected relatives had a first‐degree relative with a psychotic illness, although 8 (32%) did not have a proband participating in this study.

### Mismatch Negativity Group Differences

**Figure 3:**
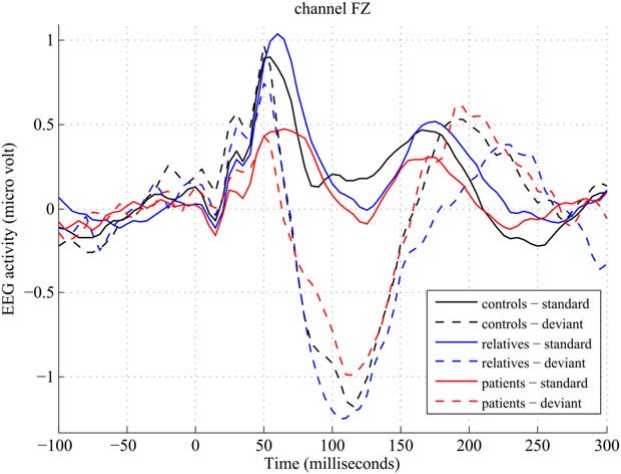
EEG activity to standard and deviant tones for each group (grand averages across subjects), at channel FZ. [Color figure can be viewed in the online issue, which is available at http://wileyonlinelibrary.com.]

The grand averaged event related potential waves for patients, relatives, and controls are shown in Figure 3. Group differences in the amplitude of the MMN wave of this sample have been reported in a previous paper [Bramon et al., 2004]: Patients with psychosis had significantly reduced MMN amplitude compared to both relatives and controls. The relatives did not differ significantly in MMN amplitude compared to the controls.

### Dynamic Causal Modeling Results

**Figure 4:**
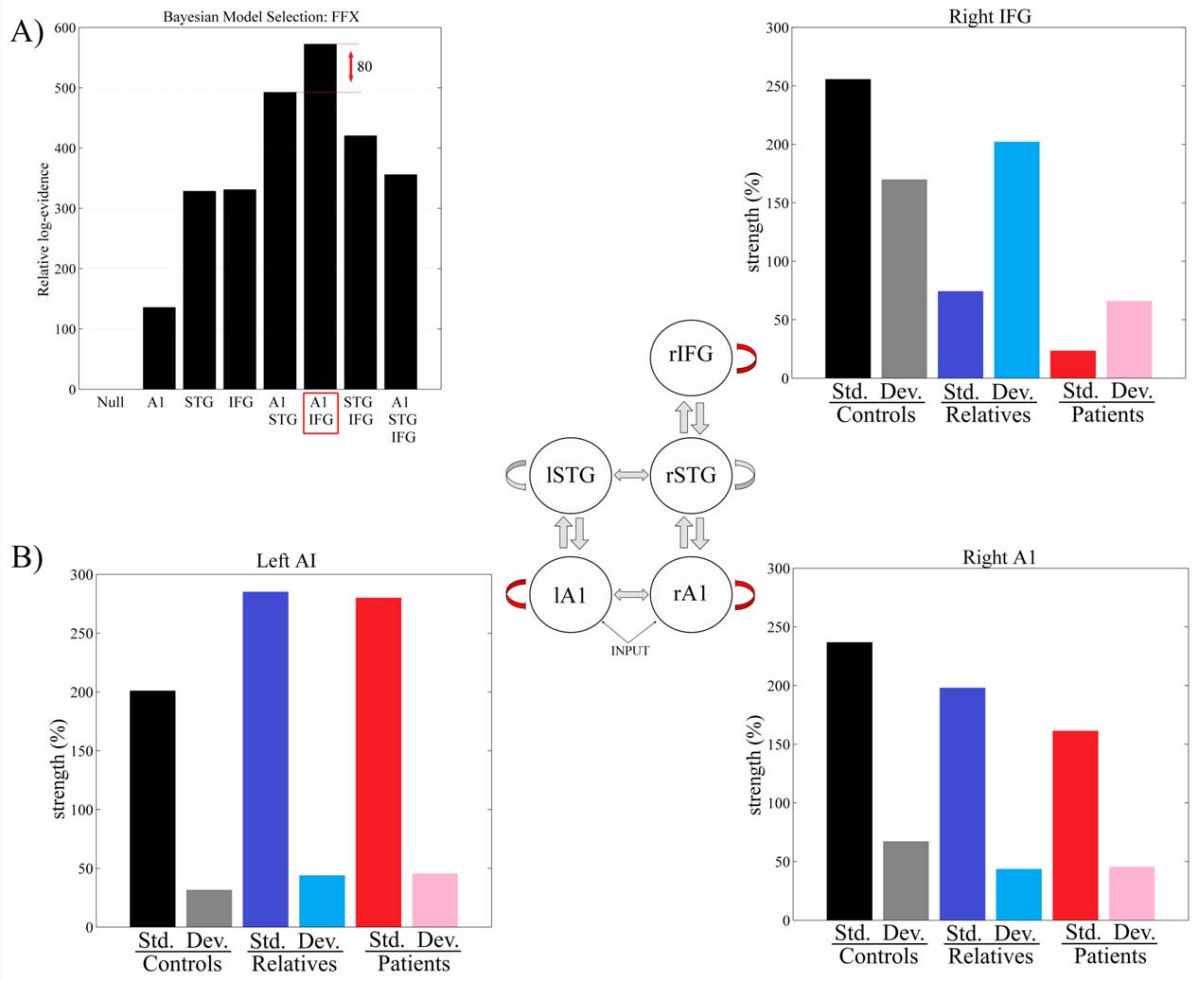
(A) Bayesian model selection results investigating intrinsic (inhibitory) modulations at different levels of the hierarchy. Log model evidences relative to the null model are shown. The winning model has modulations at A1 and IFG, and the difference in log evidence between this and the runner‐up is 80. (B) Changes in intrinsic connectivity strengths under the winning model, at each source, for patients, relatives and controls, and for standard (std.) and deviant (dev.) trials. A1 = primary auditory cortex; STG = superior temporal gyrus; IFG = inferior frontal gyrus; l = left hemisphere; r = right hemisphere. [Color figure can be viewed in the online issue, which is available at http://wileyonlinelibrary.com.]

The Bayesian model selection results are presented in Figure 4A, showing model evidences relative to the null model (with no intrinsic modulations). The model that best explained the differences between groups allowed modulations of intrinsic connectivity in bilateral A1 and rIFG. The difference in model evidence between the winning model and the runner‐up was 80. This is significant seeing as a difference of 3 (corresponding to an odds ratio of 20:1) is considered strong evidence in favour of the winning model [Friston and Penny, 2011].

**Figure 5:**
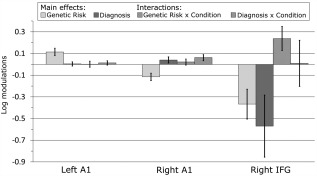
Posterior estimates of the (log scaling of) intrinsic connection parameters and their 95% confidence intervals, for each source and experimental effects investigated. A1 = primary auditory cortex; IFG = inferior frontal gyrus.

Figure 4B shows the posterior estimates of the modulations of intrinsic connectivity in the winning model for each group (controls, relatives, and patients) and condition (standard and deviant trials). Note that because the intrinsic self‐connectivity is inhibitory, increased values correspond to reduced neural excitability, and vice versa. Posterior estimates of the modulations are also shown in Figure 5, for each source and experimental effect.

The largest effects are observed at the high‐level frontal source (rIFG), where there are striking group differences. First, both relatives and patients show reduced self‐inhibition (increased excitability) across task conditions compared to controls (i.e., a main effect of having a genetic risk for psychosis). Second, patients with psychosis show an additional reduction in self‐inhibition compared to relatives, across task conditions (i.e., a main effect of diagnosis).

Third, there is a clear interaction between having a genetic risk for psychosis and task condition in rIFG; both relatives and patients show the opposite pattern of responses to the task compared to controls. While controls demonstrate reduced inhibition (i.e., increased excitability) in response to deviants compared to standard tones, the two groups with a genetic risk showed decreased excitability in response to changes in stimulus regularities.

At the sensory level (left and right primary auditory cortices, A1), all three groups show similar responses to the MMN task conditions: Increased excitability in response to deviant compared to standard tones.

## DISCUSSION

The aim of this study was to investigate whether, compared to controls, patients with psychosis and/or their unaffected relatives show altered cortical gain control (intrinsic connectivity) within cortical sources using the mismatch negativity (MMN) paradigm. We used DCM, where intrinsic connectivity is a parameterisation of the (to some extent NMDA‐R mediated) excitability of superficial pyramidal cells, which is thought to be abnormal in psychosis [Stephan et al., 2006].

Our main findings were that; (i) the largest differences in cortical responses between controls and the other groups were expressed at the top of the cortical hierarchy in the right inferior frontal gyrus (rIFG), rather than in primary sensory areas (A1); (ii) in rIFG, both groups with an increased genetic risk for psychosis (patients and their relatives) demonstrated an increase in cortical excitability across task conditions (with an additional increase in patients compared to relatives); and (iii) the two groups with a genetic risk for psychosis also showed a *reversal* of the normal pattern of increased excitability to deviant tones in rIFG.

Our finding of reduced self‐inhibition within rIFG across task conditions in those with a genetic risk for psychosis—as well as an additional reduction in patients with psychosis compared to relatives—is in line with theories of NMDA‐R hypofunction in psychosis [Abi‐Saab et al., 1998; Corlett et al., 2011; Goff and Coyle, 2001; Olney et al., 1999; Stephan et al., 2006]. Specifically, NMDA‐R hypofunction on parvalbumin positive inhibitory interneurons results in decreased inhibitory γ‐aminobutyric acid (GABA) input to (and therefore disinhibition of) pyramidal cells and hence a loss of balance between excitation and inhibition in prefrontal cortex [Lewis et al., 2012; Murray et al., 2014; Pinotsis et al., 2014]. These abnormalities may be linked to neurophysiological disorganisation [Díez et al., 2014], cognitive dysfunction and the development of symptoms of psychosis [Ahn et al., 2011; Lewis et al., 2008; Spencer et al., 2004].

Crucially, patients with psychosis and relatives show the opposite pattern of rIFG responses to deviant and standard tones, compared to controls. Controls show reduced self‐inhibition (increased excitability) in response to deviants, whereas both patients and relatives show a reduction in excitability in this condition. This indicates that those with an increased genetic risk for psychosis (including both relatives and patients) fail to adjust or optimise the excitability of superficial pyramidal cells in response to changes of stimulus regularities.

In a visual target detection task, in which subjects had to respond to target appearances that were either predictable or unpredictable, Fogelson et al. [2014] also investigated differences in intrinsic connectivity in patients with schizophrenia and healthy controls using EEG and DCM. They found that changes in intrinsic self‐inhibition in response to predictable stimuli were significantly attenuated in patients; this is further evidence that patients with schizophrenia fail to adjust neuronal connectivity in response to the context of incoming stimuli.

Our results can be interpreted in the context of predictive coding theories of brain function, in which the brain infers the causes of its sensory data using Bayesian inference by minimizing prediction errors throughout the cortical hierarchy [Friston, 2008; Rao and Ballard, 1999]. Predictive coding can be implemented neurobiologically by deep pyramidal cells sending top‐down predictions about lower level representations, and superficial pyramidal cells sending bottom‐up prediction errors (the difference between the actual and predicted activity) back up the hierarchy, in order to update the higher level representations [Friston, 2008]. These neurobiological details are important, because superficial pyramidal cells—that is, prediction error units—make the primary contribution to event related potentials [Garrido et al., 2009b; Lieder et al., 2013]. Crucially, the influence of ascending prediction errors on higher representations depends upon their precision, which is thought to be encoded by the gain or excitability of superficial pyramidal cells. In this setting, precision (inverse variance) corresponds to the confidence or reliability attributed to prediction errors at each level of the cortical hierarchy [Adams et al., 2013; Feldman and Friston, 2010].

In our MMN data, controls show increased synaptic gain (diminished intrinsic self‐inhibition) in all cortical sources in the deviant condition—that is, their prediction error responses to deviant tones are processed as being unduly precise and are therefore less easily suppressed. This is also the case for all individuals with a genetic risk for psychosis at the primary sensory level, but in rIFG the opposite pattern is seen. This indicates an abnormal influence of context on prediction error responses in this group, as has been seen not only in perceptual paradigms like the MMN, but also in reward learning and causal inference paradigms [Corlett et al., 2007; Murray et al., 2008].

In computational modeling work, we have shown that a loss of precision at higher levels of a hierarchical model can explain a loss of influence of context [Adams et al., 2013]. Predictive coding simulations show that aberrant precision or gain control can reproduce classic findings in the schizophrenia literature, including a reduced MMN response [Adams et al., 2013]. NMDA‐R hypofunction could confound precision or gain control in two ways, either by directly lowering synaptic gain in superficial pyramidal cell populations, or by reducing the excitability of GABAergic interneurons, thereby impairing sustained oscillatory firing of pyramidal cells and reducing their influence on lower areas [Adams et al., 2013]. Our current results lend more support to the latter mechanism, and it would be interesting to test this hypothesis directly by using DCM to assess the relative model evidences for psychosis altering the excitability of superficial pyramidal cell versus inhibitory interneuron populations.

Importantly, our results suggest that both patients and their first degree relatives have similar alterations in the excitability of superficial pyramidal cell populations, compared to controls. This indicates that these changes are linked to genetic risk factors, and are not merely a consequence of the illness state or antipsychotic medication. This alteration in the gain of superficial pyramidal cells could therefore be a potential endophenotype for psychosis [Gottesman and Gould, 2003]. The use of endophenotypes might help clarify the functional effects of genetic risk variants identified [Bramon et al., 2014; Hall and Smoller, 2010], and further research could investigate whether deviant‐related changes in excitability can predict genotype; for example, looking at candidate genes linked to NMDA‐R function. Other studies investigating effective connectivity in psychosis have also observed abnormalities in relatives of patients, including children of probands [Diwadkar et al., 2014, 2012; Winterer et al., 2003], and a previous study by Dima et al [2013] observed associations between fMRI derived measures of effective connectivity and risk genes linked to GABAergic interneuron function in patients with bipolar disorder.

Our results also suggest that patients show a further increase in excitability in rIFG across task conditions compared to unaffected relatives. This may indicate that—at least in prefrontal cortex—there are quantitative, rather than qualitative, differences between those with and without a diagnosis of a psychotic illness but at elevated genetic risk. Alternatively, this difference could be due to the effects of antipsychotic medication, which is known to influence brain function [e.g., Joutsiniemi et al., 2001; Knott et al., 2001]. The exact effects of psychotropic drugs on effective connectivity remain unclear; however, a study investigating effective connectivity in schizophrenia found abnormalities in an unmedicated at‐risk group but not in first episode patients (prescribed antipsychotics), suggesting that medication might potentially normalise abnormalities [Schmidt et al., 2013]. Future longitudinal studies and research in unmedicated patient populations are needed to address this important issue.

A limitation of the current study is that our groups differed slightly in age and gender distributions. There is evidence for both age [Cooper et al., 2006; Cooray et al., 2014; Kiang et al., 2009; Näätänen et al., 2012] and gender [Brossi et al., 2007; Matsubayashi et al., 2008] effects on MMN responses, although a DCM study did not find significant effects of aging on intrinsic connectivity [Moran et al., 2014]. Importantly, however, we found the most significant effects when comparing those with a genetic risk for psychosis (i.e. both relatives and patients) with controls, and since these two groups did not differ in age or gender distributions, our main findings are unlikely to be influenced by such confounds.

Another potential limitation is the experimental procedure used to elicit the MMN response. Because the MMN is a preattentive response not depending on the person paying attention to the sounds, it has been suggested that using a distractor task (such as watching a silent video or reading a book) can be advantageous [Duncan et al., 2009; Lang et al., 1995]. In this study, no distractor task was administered, and participants were instructed to disregard the sounds presented to them. We can therefore not control whether participants were paying attention to the task or not. Nevertheless, this distractor‐free design has been used previously and has been shown to generate clear MMN responses [Bramon et al., 2004; Haenschel et al., 2000; Javitt et al., 1998; Juckel et al., 2007]. Furthermore, attention has been found to modulate the MMN response suggesting this ERP might not actually be independent of attention [Auksztulewicz and Friston, 2015; Sussman et al., 2013; Woldorff et al., 1991].

Our Bayesian model selection result indicates that both bilateral A1 and rIFG are important in explaining group differences in modulations of intrinsic connectivity in response to deviant tones. However, modulations of self‐inhibition in STG do not seem to be so important (and were not included in the winning model). Importantly, this does not mean that the STG makes no contribution to group differences in responses, but merely suggests that including modulations in this region did not increase the evidence for the model sufficiently to justify the increased complexity. Our results furthermore suggest that group differences are most pronounced in rIFG. This is in line with past research suggesting that psychosis is associated with abnormalities at high hierarchical levels, including the prefrontal cortex [reviewed in Adams et al., 2013; Harrison et al., 2011].

We chose to calculate condition‐specific grand average responses for each group, an approach that has been used previously [e.g., Fogelson et al., 2014]. While this produces cleaner data features by reducing noise and enhancing features that are conserved over subjects, it eliminates potentially interesting individual differences. Future work could obtain subject‐specific DCM estimates, allowing the investigation of individual differences within groups, and correlations between effective connectivity parameters and various clinical and cognitive measures, as well as with genotypes.

## CONCLUSION

In summary, our main finding is that patients with psychosis as well as their unaffected first‐degree relatives show increased excitability in rIFG across task conditions, relative to controls, and crucially, a loss (reversal) of the normally increased excitability in deviant trials. Hence, our results suggest that psychosis is associated with abnormalities of the sensitivity (gain) control of superficial pyramidal cell populations, which might be influenced by NMDA‐R hypofunction in prefrontal cortex. These results are in line with theories about the neuropathology and pathophysiology of psychosis. Importantly, abnormalities in unaffected relatives of patients suggest that these alterations are linked to the aetiology of psychosis and are potential endophenotypes (markers of genetic risk) for the illness.

## Supporting information

Supporting Information
