# Supplementary material for: Impaired prefrontal synaptic gain in people with psychosis and their relatives during the mismatch negativity
Source: Hum Brain Mapp. 2015 Oct 27;37(1):351–65. doi: 10.1002/hbm.23035 (PMC4843949; doi:10.1002/hbm.23035)
Supplement: Supplementary file 1 — Supporting Information [file HBM-37-351-s001.docx]

# Supplementary material


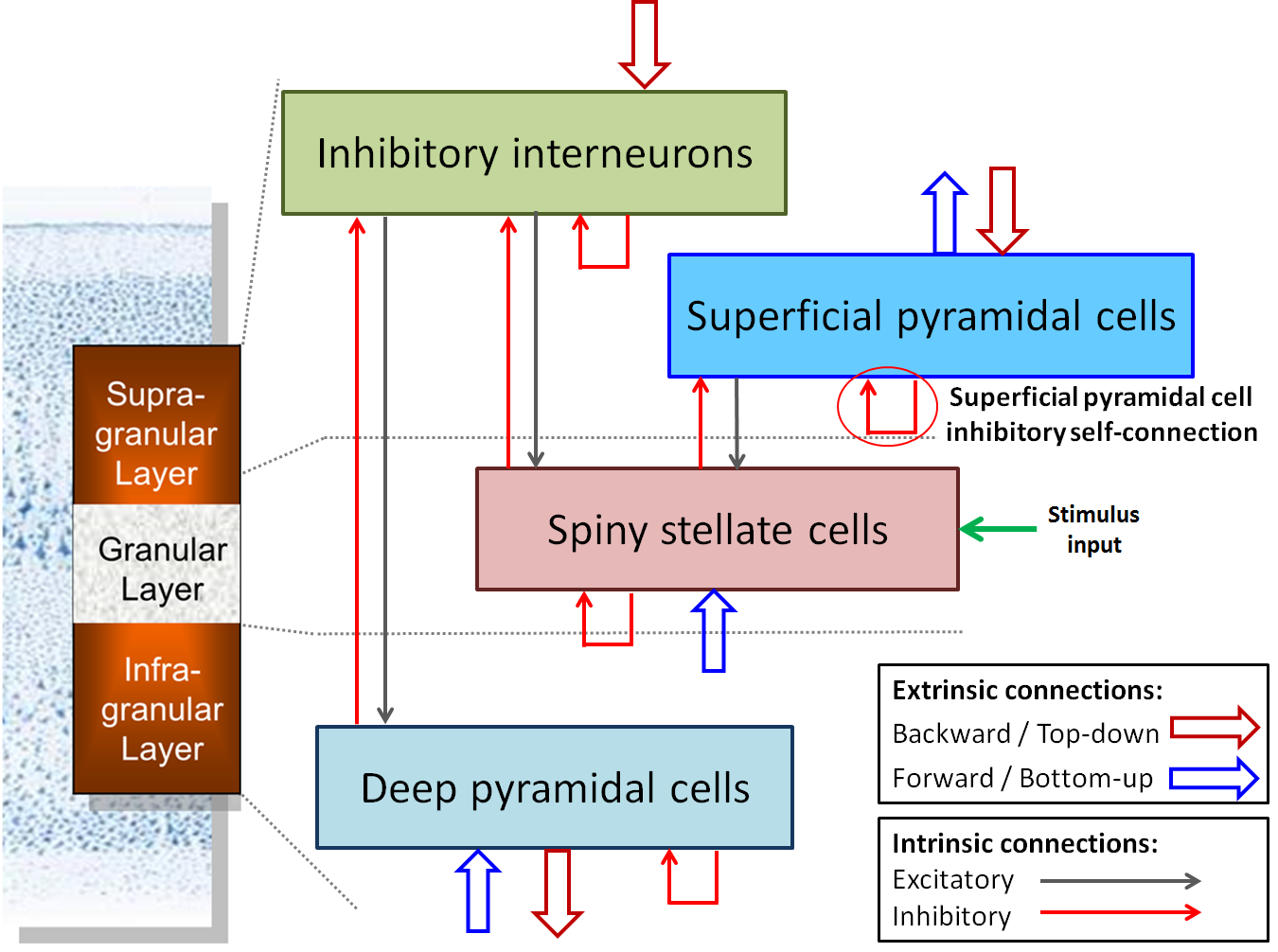


Figure S1. The Canonical Microcircuit neural mass model, showing the four cell populations within each cortical source, and the extrinsic and intrinsic connections. In this study, we focused on the self-inhibition of superficial pyramidal cells (circled).

| 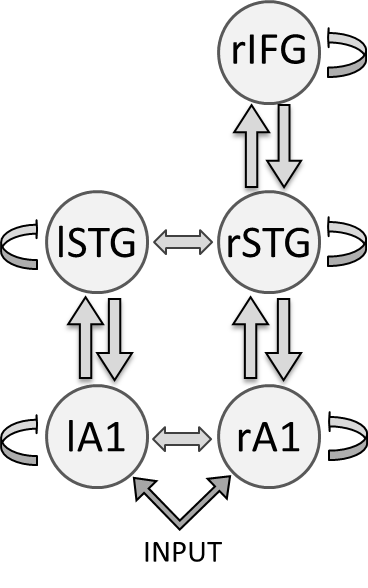 | 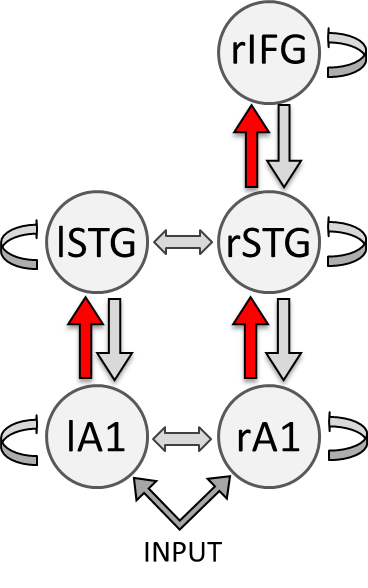 | 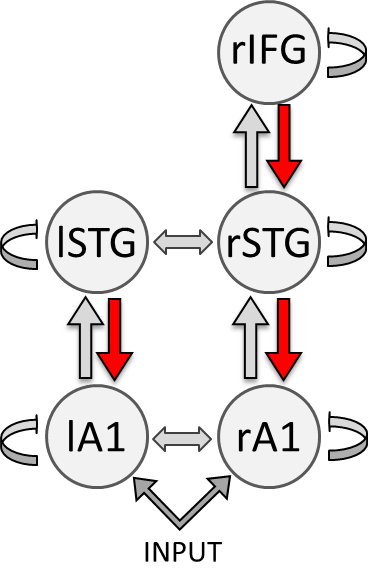 | 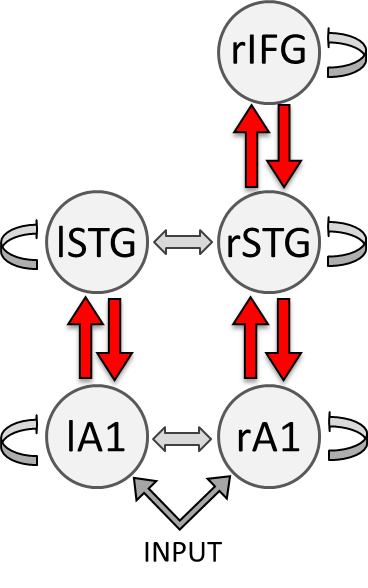 |
| --- | --- | --- | --- |
| **null** | **F** | **B** | **FB** |
| 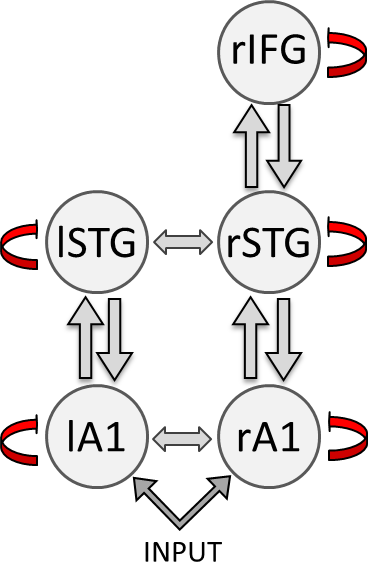 | 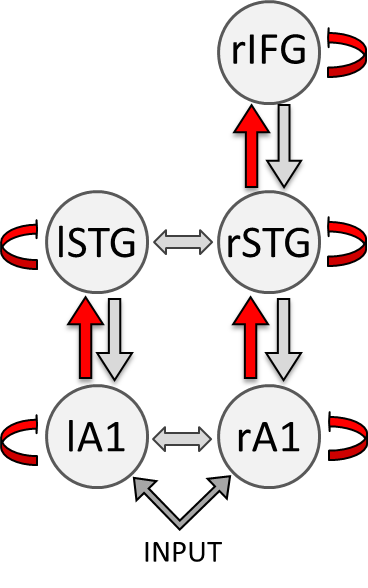 | 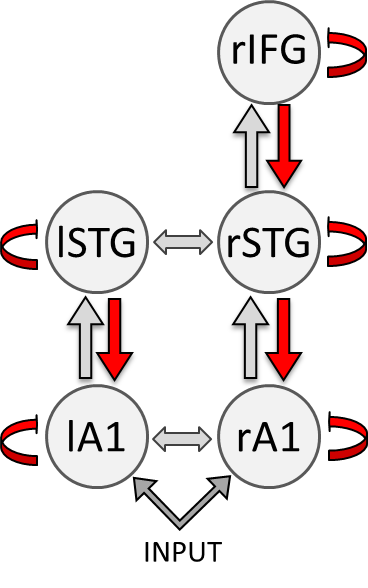 | 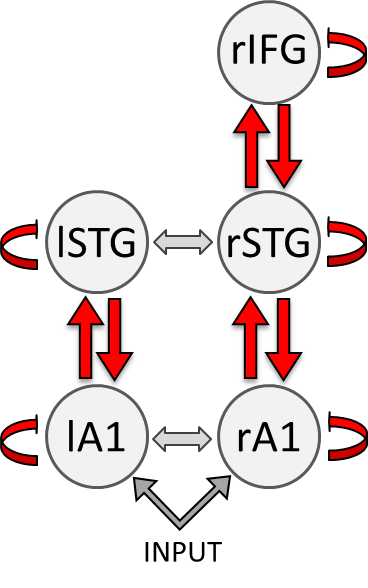 |
| **I** | **FI** | **BI** | **FBI** |

Figure S2. Dynamic causal modelling (DCM) model space, investigating the mismatch negativity responses across the three groups. Red arrows indicate a modulated connection. A1 = primary auditory cortex; STG = superior temporal gyrus; IFG = inferior frontal gyrus; l = left hemisphere; r = right hemisphere. F = forward; B = backward; I = intrinsic.

Figure S3. Bayesian model selection results investigating differences between standard and oddball tones across the three groups. F = forward modulations; B = backward modulations; I = intrinsic modulations.
